# Evaluation of Transmitral Pressure Gradients in the Intraoperative Echocardiographic Diagnosis of Mitral Stenosis after Mitral Valve Repair

**DOI:** 10.1371/journal.pone.0026559

**Published:** 2011-11-08

**Authors:** Ann K. Riegel, Raila Busch, Scott Segal, John A. Fox, Holger K. Eltzschig, Stanton K. Shernan

**Affiliations:** 1 Department of Anesthesiology, University of Colorado Denver, Denver, Colorado, United States of America; 2 Department for Cardiology, Angiology, Pneumology and Intensive Care Medicine, University Hospital Greifswald, Greifswald, Germany; 3 Department of Anesthesiology, Tufts Medical Center, Boston, Massachusetts, United States of America; 4 Department of Anesthesiology, Perioperative and Pain Medicine, Brigham and Women's Hospital, Harvard Medical School, Boston, Massachusetts, United States of America; University of Colorado Denver, United States of America

## Abstract

**Objective:**

Acute mitral stenosis (MS) following mitral valve (MV) repair is a rare but severe complication. We hypothesize that intraoperative echocardiography can be utilized to diagnose iatrogenic MS immediately after MV repair.

**Methods:**

The medical records of 552 consecutive patients undergoing MV repair at a single institution were reviewed. Post-cardiopulmonary bypass peak and mean transmitral pressure gradients (TMPG), and pressure half time (PHT) were obtained from intraoperative transesophageal echocardiographic (TEE) examinations in each patient.

**Results:**

Nine patients (9/552 = 1.6%) received a reoperation for primary MS, prior to hospital discharge. Interestingly, all of these patients already showed intraoperative post-CPB mean and peak TMPGs that were significantly higher compared to values for those who did not: 10.7±4.8 mmHg vs 2.9±1.6 mmHg; p<0.0001 and 22.9±7.9 mmHg vs 7.6±3.7 mmHg; p<0.0001, respectively. However, PHT varied considerably (87±37 ms; range: 20–439 ms) within the entire population, and only weakly predicted the requirement for reoperation (113±56 vs. 87±37 ms, p = 0.034). Receiver operating characteristic curves showed strong discriminating ability for mean gradients (AUC = 0.993) and peak gradients (area under the curve, AUC = 0.996), but poor performance for PHT (AUC = 0.640). A value of ≥7 mmHg for mean, and ≥17 mmHg for peak TMPG, best separated patients who required reoperation for MS from those who did not.

**Conclusions:**

Intraoperative TEE diagnosis of a peak TMPG ≥17 mmHg or mean TMPG ≥7 mmHg immediately following CPB are suggestive of clinically relevant MS after MV repair.

## Introduction

Mitral valve repair has become the procedure of choice for patients with significant MV dysfunction of most etiologies [1], [2]. Repair of the MV is reportedly superior to replacement since it is associated with better preservation of valve tissue, subvalvular apparatus and left ventricular function, as well as improved long-term survival [3], [4]. Furthermore, MV repair permits greater protection from endocarditis, thomboembolism and anticoagulation-related morbidity [5], [6].

Recurrent mitral regurgitation (MR) is well recognized as the most common cause for failure of MV repair [7], [8]. Another less common complication is the development of late mitral stenosis after MV repair especially for rheumatic disease [9] but also after MV repair for non-rheumatic MR [10]. Ibrahim *et al.* reported a 1% incidence of late MS, manifesting 3–9 years after MV repair for non-rheumatic MR [10]. Direct inspection of the MV repair in patients who underwent reoperation in this study revealed hindered, free leaflet motion associated with pannus formation on the anuloplasty ring[10]. In contrast to the development of late MS there is little information available on acute MS immediately following MV repair. In a case study, Maslow et al. reported the occurrence of a mitral stenosis immediately after mitral valve repair in a 37 year old female patient with myxomatous mitral valve disease [11]. In addition, an earlier study of Muratori et al. reported an incidence of a single case of intraoperatively diagnosed acute mitral stenosis out of a group of 142 patients also with myxomatous mitral valve disease who underwent mitral valve repair [12]. However, systematic reports of acute MS after MVP in larger cohorts of patients have not been published.

Echocardiography is commonly used to diagnose and quantify primary, native MS. Well established diagnostic criteria include amongst others planimetry, gradient measurements and estimation of pressure half-times [13]. However, alterations in MV orifice geometry following repair, or changes in chamber compliance after cardiopulmonary bypass (CPB) were shown to influence the intra- and postoperative echocardiographic evaluation of MS [14], [15]. The calculation of mitral valve area by pressure half time measurements immediately after mitral valve repair was shown to underestimate the actual mitral valve area [15]. This led to the question which echocardiographic indices of MS severity still provide reliable information in the intraoperative setting, since specific echocardiographic criteria for the diagnosis of acute MS after MV repair have not been well established.

Intraoperative echocardiography is commonly used in the management of cardiac surgical patients [16], [17]. In fact, intraoperative echocardiographic diagnosis of MS following MV repair would be desirable, since it would permit prompt surgical revision before the development of postoperative morbidity and mortality. Therefore, we retrospectively analyzed the medical records and intraoperative, transesophageal (TEE) Doppler echocardiographic examinations of patients undergoing MV repair for MR, to determine specific echocardiographic criteria for defining significant acute MS.

## Methods

### Patient Population

The study population consisted of all patients undergoing MV repair for MR at the Brigham and Women's Hospital between 2001 and 2003 of whom a post-CPB, transmitral Doppler flow velocity profile was obtained and recorded for off-line analysis. 247 patients out of 552 were diagnosed with ischemic MR, 164 with myxomatous degenerative mitral valve disease, 27 with rheumatic heart disease and 17 patients were diagnosed with endocarditis leading to MR. All patients were consented for an intraoperative TEE during preoperative interview. Consent was given in written form. The approval for this retrospective study was obtained from the Institutional Review Board, Brigham and Women's Hospital, to review the patients' medical records and intraoperative TEE examination reports.

### Echocardiographic Data

Comprehensive intraoperative TEE examinations were performed using multiplane probes (Siemens, Mountain View, CA; Philips Healthcare, Inc, Andover, MA). All TEE examinations were performed by cardiac anesthesiologists with extensive experience in perioperative echocardiography. Peak and mean TMPGs were determined using the simplified Bernoulli Equation from either pulse wave Doppler flow velocities (PWD) obtained at the tips of the mitral leaflets, or continuous wave Doppler to identify transmitral velocities when aliasing occurred despite optimal adjustment of the scale and baseline. TEE examinations were recorded on super VHS tape and analyzed off-line by a cardiac anesthesiologist (H.K.E.) and a cardiologist (R.B.) with extensive experience in perioperative echocardiography. Both examiners were blinded to the clinical outcome data. Analysis of the echocardiographic data included calculations of the peak and mean TMPG, and PHT from the post-CPB transmitral Doppler flow velocity profiles. Values for mean and peak TMPG and PHT were obtained from the average of three separate measurements.

### Decision to return to CPB to re-do the mitral valve repair

The decision to return to CPB to revise the original MV repair or replace the valve was made on an individual basis for each patient and included the following standard considerations: (a) the degree of hemodynamic instability (b) the patient's co-morbidity (c) potential additional morbidity associated with a prolonged second period of CPB (d) the surgeons' opinion as to their ability to produce a better result (e) echocardiographic findings, particularly from 2D echocardiography suggestive of MS (e.g. restricted leaflet mobility). Leaflet restriction was reported, but not objectively quantified, by the cardiac anesthesiologists who performed the intraoperative TEE examination. While echocardiographic measurements of TMPGs were available, cut-off values indicating significant acute iatrogenic MS following MV repair were not known at the time of this study.

### Review of Medical Records and Follow-Up

Medical records were reviewed for patients' demographics, type of surgical procedure and MV repair, and the incidence and indication for MV reoperation prior to hospital discharge.

### Statistical Analyses

Demographic data were tabulated and descriptive statistics calculated. The echocardiographic data from the two independent analyses were averaged. Interobserver variability was assessed with Pearson correlation, and r and 95% confidence interval were calculated. Mean values for echocardiographic parameters were compared by unpaired t-test. Receiver operating characteristic (ROC) curves, area under the curve (AUC) and standard error (SE) were calculated with the use of the Graph Pad Prism 5 software. Values for best discrimination of cases requiring and not requiring reoperation were estimated by inspection. When exact P values were not specified, P<0.05 was considered significant.

## Results

### Patient Population

A total of 552 patients who underwent MV repair were included in the analysis. An additional 26 patients did not have interpretable Doppler recordings. Demographic data, type of surgical procedure and a description of the MV repair are displayed in Table 1. Nine (9/552 = 1.6%) patients with intraoperative TEE evidence of restricted MV leaflet motion underwent reoperation for MS prior to hospital discharge, including 4 patients who underwent surgical revision of the initial MV repair immediately following the post-CPB echocardiographic examination (Table 2). All of these patients were receiving inotropic and pressor support while attempting to wean from CPB following MV repair. None of these patients demonstrated significant concurrent MR.

**Table 1:** Patient Characteristics and Surgical Procedure (N = 552).

**Age**	63.3±14.1	
**Gender**	188 F/364 M	
**Surgical procedure**		
	**Primary**	**Reoperation**
MV Repair	203	10
MV Repair + CABG	226	20
MV Repair + other valve (AVR, TVR)	45	15
MV Repair + other valve (AVR, TVR) + CABG	33	0
**Type of Repair**		
Isolated Annuloplasty	453	
Annuloplasty + Leaflet Resection	6	
Annuloplasty + Chordal Repair	1	
Annuloplasty + Commisurotomy	1	
Annuloplasty + Maze procedure	1	
Annuloplasty + Pericardial Patch	1	
Isolated Alfieri (“edge-to-edge”)	29	
Alfieri (“edge-to-edge”) + Leaflet Resection	1	
Alfieri (“edge-to-edge”) + Commisurotomy	1	
Alfieri (“edge-to-edge”) + Chordal Repair	1	
Alfieri (“edge-to-edge”) + Ring Annuloplasty	49	
Ring Annuloplasty + Leaflet Resection + Alfieri Stitch	3	
Other	5	
**Rings used for annuloplasty**		
Carbomedics	12	
Carpentier-Edwards	181	
Cosgrove-Edwards	341	
Duran	7	
Medtronic	1	
No ring	37	

yr: years; M/F; Male/Female; CABG: coronary artery bypass grafting, MV: mitral valve, AVR: aortic valve replacement, TVR: tricuspid valve repair.

**Table 2:** Characteristics of Patients with Mitral Stenosis after Mitral Valve Repair.

Age	M/F	MR Etiology	Primary Procedure	P/M (mmHg)	Reoperation Time	Reoperation Type	LOS (d)	D/C y/n
67	M	Ischemic MR	CABG, AVR, Alfieri	22/12	Post CPB	Alfieri Revision	13	y
54	F	Myxomatous	# 38 C-E-P	38/22	Post CPB	MVP Revision	7	y
39	M	Myxomatous	# 30 CG	19/7	2 d	# 36 CG	6	y
69	M	Myxomatous	# 28 CG, CABG	17/7	Post CPB	MVR # 29 Hancock	10	y
36	F	Myxomatous Endocarditis	# 34 CG, Alfieri	18/10	1 d	MVR # 31 St Jude	22	y
76	F	Ischemic MR	# 26 C-E-P	21/11	Post CPB	MVR # 27 C-E-P	20	y
52	F	Myxomatous	# 26 C-E-P, Alfieri	31/12	8 d	# 28 CM Annuflex Ring	15	y
77	F	Myxomatous	# 28 MT Ring	17/7	6 d	MVR #29 Hancock	7	y
54	F	Myxomatous	#32 CG, Alfieri	19/7	12 h	Alfieri Revision	7	y

M/F: Male/Female; MR: mitral regurgitation; P/M: peak and mean transmitral mitral pressure gradients obtained by post-CPB, intraoperative transesophageal echocardiography; LOS: length of hospital stay; d:days; h:hours; D/C: discharge from hospital; y: yes; n: no; MVP: mitral valve repair; MVR: mitral valve replacement; CPB: cardiopulmonary bypass; AVR: aortic valve replacement; CABG: coronary artery bypass grafting; CG: Cosgrove-Edwards annuloplasty; C-E-P: Carpentier Edwards ring annuloplasty; MT: Medtronic; CM: Carbo Medics.

### Interobserver Variability

Interobserver variability was excellent for both measures of TMPG: Pearson's r and 95% CI were 0.989 (0.987, 0.991) for peak gradient and 0.964 (0.958, 0.970) for mean gradient. PHT correlated less well between observers (r = 0.263 [0.183, 0.340]). All correlations were highly significant (P<0.0001).

### Transmitral Pressure Gradients and Pressure Half-Time

**Figure 1:**
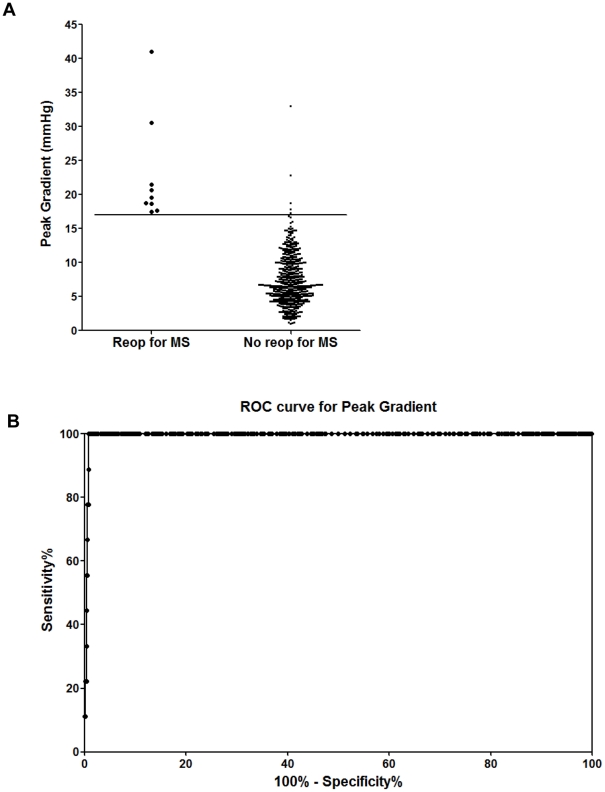
Distribution of peak gradients, split by the requirement for reoperation for mitral stenosis (MS). (A) A peak gradient of ≥17 mm Hg, best separated cases requiring reoperation for MS from those that did not. (B) Receiver operator curves (ROC) for peak transmitral pressure gradients. The area under the curve (AUC [SE]) for peak transmitral pressure gradients (0.996 [0.003]) showed strong discriminating ability.

**Figure 2:**
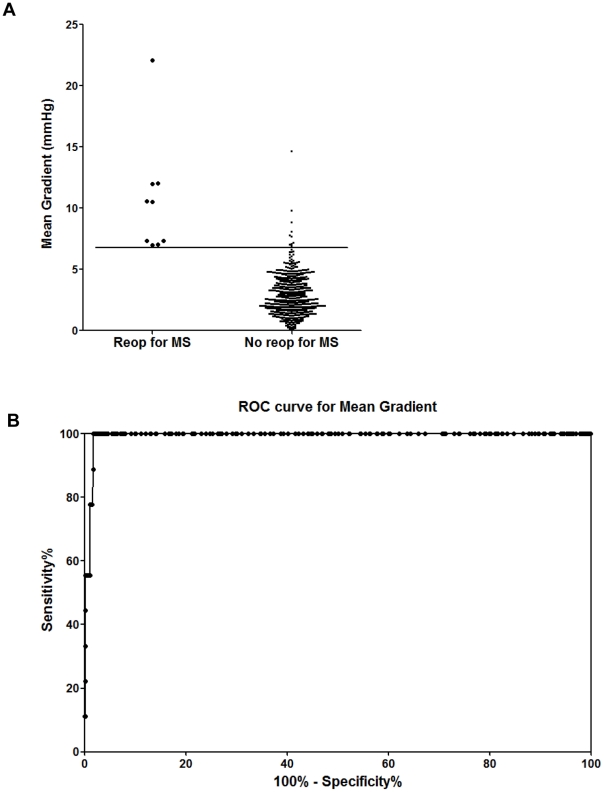
Distribution of mean gradients, split by the requirement for reoperation for mitral stenosis (MS). (A) A mean gradient of ≥7 mm Hg, best separated cases requiring reoperation for MS from those that did not. (B) Receiver operator curves (ROC) for mean transmitral pressure gradients. The area under the curve (AUC [SE]) for mean transmitral pressure gradients (0.993 [0.003]) showed strong discriminating ability.

**Figure 3:**
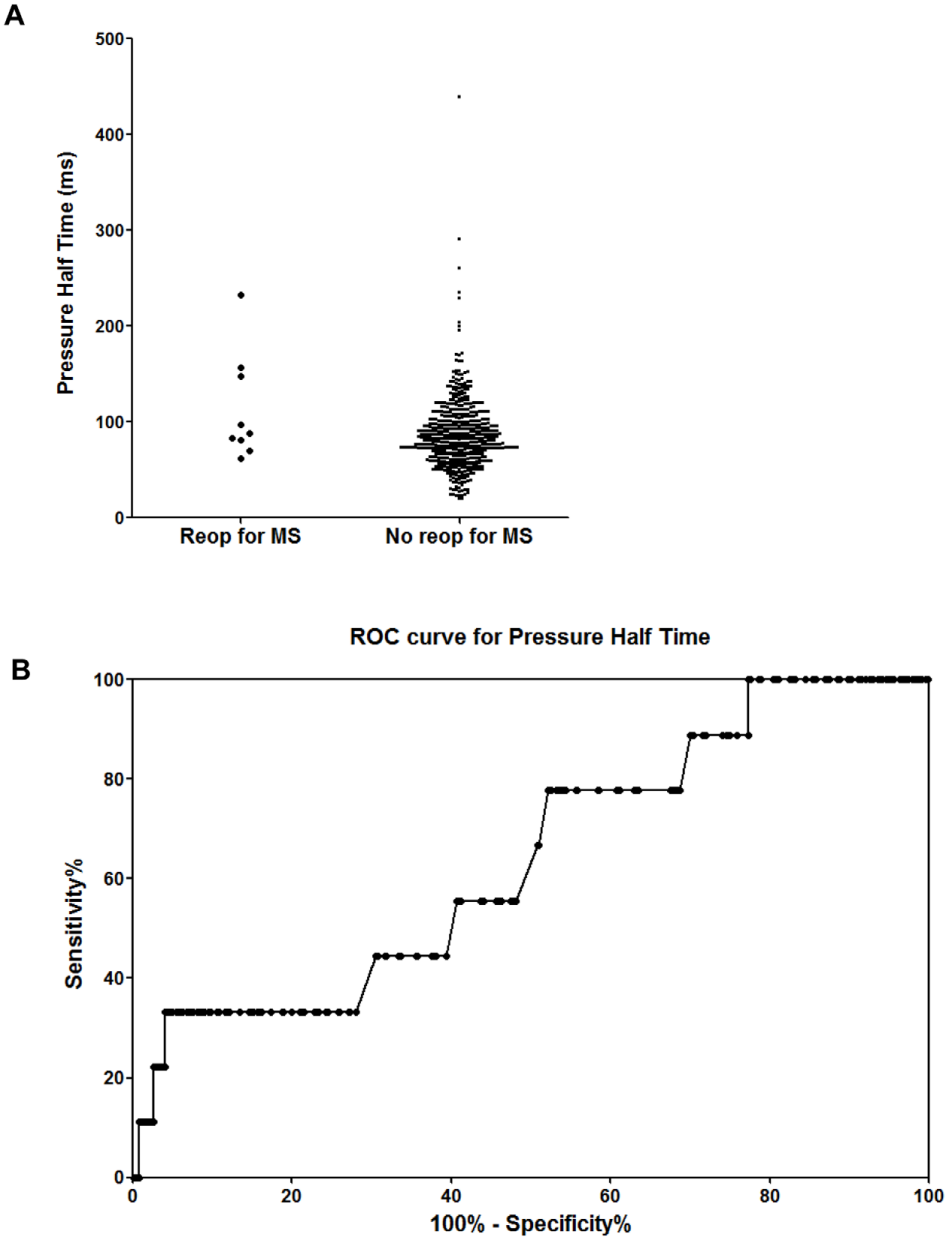
Distribution of pressure half times after mitral valve repair. (A) Distribution of pressure half times showed no significant difference in distribution between cases requiring reoperation for MS and those that did not. (B) Receiver operator curve (ROC) for pressure half time (PHT). The area under the curve (AUC [SE]) showed only weak discriminating ability (0.640 [0.092]).

Mean and peak postoperative TMPGs for the entire population (mean ± SD) were 3.1±2.0 mmHg and 7.6±4.2 mmHg respectively. Patients with restricted MV leaflet motion by post-CPB intraoperative TEE who did not have persistent significant MR and required a MV reoperation for MS, had a mean TMPG of 10.7±4.8 mmHg vs. 2.9±1.6 mmHg without MS and had a peak TMPG of 22.9±7.9 mm Hg vs. 7.6±3.7 mmHg without MS (P<0.0001 for each comparison) measured by intraoperative TEE. All of these patients were discharged from the hospital. PHT varied considerably (87±37 ms; range: 20–439 ms) and only weakly predicted a requirement for reoperation (113±56 vs. 86±37 ms, P = 0.034). ROC curves for peak and mean TMPG and PHT are shown in Figures 1, 2, and 3, respectively. The AUC (SE) for pressure gradients showed strong discriminating ability for both peak TMPG (0.996 [0.003]) and mean TMPG (0.993 [0.003]), but much weaker ability for PHT (0.640 [0.092]).

Values for separation of cases requiring reoperation for MS from those that did not were estimated by inspection of the distribution of peak and mean TMPG values (Figure 3). A peak gradient of ≥17 mmHg, or mean gradient of ≥7 mmHg, best separated these patients.

## Discussion

The development of MS following MV repair is most commonly associated with late degenerative changes and fibrous overgrowth, which restrict diastolic leaflet excursion over the time [10], [18]. In contrast, acute MS following with mitral valve repair surgery can present intraoperatively, immediately after the termination of CPB. The exact incidence however remains unknown, as previous reports are either single case reports [11] or are based only on small numbers of patients [12] and studies in different centers might be highly influenced of patient heterogeneity. Here, we used intraoperative TEE to identify a peak or mean TMPG of at least 17 mmHg or 7 mmHg, respectively, as indicators of significant early MS in 9 out of 552 patients who subsequently required prompt surgical revision following an initial MV repair for primary MS. All of these patients survived to be discharged from the hospital. Thus, intraoperative TEE may be useful for accurately and efficiently identifying patients with acute MS following MV repair who may benefit from a prompt surgical revision before the development of significant postoperative morbidity and mortality.

Intraoperative ultrasound and TEE is a widely used, safe and practical technique [17], [19]–[34]. TEE can be used for the intraoperative evaluation of the mitral valve [17], [35], including evaluation for MS severity [36]. However, two-dimensional echocardiographic diagnosis of MS following post-MV repair may be difficult in some patients including those undergoing an Alfieri edge-to-edge repair in which the mid-portion of the anterior and posterior leaflet are intentionally sutured together to prevent MR [37]. Interestingly, although 3 of the 9 patients in our series who required reoperation for MS initially underwent edge-to-edge repairs, others have reported that this technique can still significantly decrease MR by reducing MV area while maintaining mean TMPG<6 mmHg [37] and preserving MV reserve [38].

Classical Doppler echocardiographic measures for quantifying native MS may not be applicable immediately following MV repair due to acute changes in orifice geometry and chamber compliance [14]. Limitations in using Doppler echocardiography to assess MS severity have been described in patients undergoing mitral valvotomy. Although Hatle et al. demonstrated a reliable, inverse correlation between Doppler echocardiographic measurements of PHT and MV orifice area in patients with native MS [38], the same correlation between PHT and MV area could not be demonstrated in patients with MS immediately after mitral valvotomy [14]. Similarly, while Maslow et al. demonstrated good agreement and correlation between MV area with PHT and planimetry obtained with two-dimensional echocardiography in patients undergoing MV repair [39], others have shown that intraoperative TEE measurement of PHT following MV repair may be unreliable and can underestimate MV area [15]. In our study, PHT varied considerably and only weakly predicted a requirement for reoperation, suggesting that PHT may be dependent upon hemodynamic variables other than MV orifice area including net left atrial and ventricular compliance [14].

Estimating MV area using the PISA technique hs been demonstrated in patients with native MS, and has been used to estimate mitral regurgitant orifice area following MV surgery [40]. However, PISA has not been consistently validated for assessing acute MS immediately after MV repair. Furthermore, the estimation of MV area using the PISA technique may be relatively time consuming, and therefore impractical to apply in the immediate post-CPB period while a hemodynamically unstable patient is being resuscitated.

Finally, 3D echocardiography is a rapidly evolving technique which is increasingly used intraoperatively during mitral valvuloplasty [41] and mitral valve repair [42]. In primary, native mitral stenosis, estimation of MVA with 3D echocardiography is considered to be the gold standard of diagnosis of mitral stenosis by some authors [43]. However, until now, no study is available which examined the reliability of 3D TEE MVA measurements in identifying acute MS in the intraoperative setting immediately after MVP.

Alternatively, as demonstrated in the present study, TMPGs obtained by Doppler echocardiography are reliable measures of MS severity, highly reproducible, easy to acquire and should therefore be considered an important component of the post-CPB intraoperative echocardiographic examination in patients undergoing MV surgery.

Echocardiographic calculation of TMPG as a measure of MS severity may be influenced by the presence of concurrent MR [44]. None of the patients in our study who required reoperation for significant MS demonstrated concurrent significant MR. Conventional echocardiographic measures of MS severity may also be influenced by changes in cardiac output. Mohan et al. used dobutamine stress echocardiography in 57 ambulatory patients with MS to show that alterations in transmitral flow are associated with small and clinically insignificant changes in directly planimetered MV area, but more pronounced changes in MV as determined by PHT [45]. In addition, Firstenberg et al. also used stress echocardiography in 13 patients with MS to demonstrate that changes in cardiac output result in predictable changes in PHT [46]. Although increases in cardiac output may promote MV orifice stretching and reserve associated with decreases in PHT, increased flow rates may also be associated with higher TMPGs [46]. All patients with significant MS in our study who eventually underwent surgical revision were hemodynamically compromised and were receiving inotropic and pressor support, however intraoperative cardiac output was not routinely measured during the post-CPB echocardiographic examination. Therefore, we were unable to determine the specific influence of cardiac output on echocardiographic measures of MS severity. Nonetheless, direct and indirect echocardiographic measures of MV area appear to remain valid under conditions of varying transmitral flow [45], [46]. Finally, Doppler echocardiographic measures of MS severity may also be influenced by changes in diastolic function including impaired LV relaxation and compliance. However, all of the patients in our study with a presumed diagnosis of acute MS post MV repair had echocardiographic evidence of restricted MV leaflet motion, and furthermore, it is uncommon for peak TMPG to exceed 17 mmHg due to isolated, impaired LV compliance.

Intuitively, one might expect to see a higher prevalence of iatrogenic MS in patients undergoing MV repair for ischemic MR using a relatively restrictive annuloplasty compared to surgical approaches for repairing degenerative etiologies of MR. However, in our series, patients who underwent only annuloplasty ring placement for ischemic or functional MR seemed less susceptible to acute MS after MVP (2 out of 247 patients) perhaps due to extensive surgical experience in sizing rings. On the other hand, patients with myxomatous MV disease were more likely to require repairs that involved increased complexity associated with leaflet resection and reconstruction including edge-to-edge repairs. This might have been the reason for the increase in incidence of acute MS after MVP of myxomatous valves (6 out of 164 patients). Moreover, this might underline the benefit of intraoperative measurement of TMPGs especially during MVPs of myxomatous mitral valves.

Some important limitations of the present studies should be noted. The present findings are somewhat confounded by the availability of Doppler data in an un-blinded fashion during surgery, such that the decision to revise the original MV repair may have been partly based on the echocardiographic findings. Thus, the lack of independence between the measure and the outcome has the potential to overestimate the strength of the relationships. Secondly, transmitral flow measurements are flow dependent and the present study did not include the integration of flow measurements into the assessment of MS severity. Thirdly, despite the large number of patients that were included in the present study, only a relatively small number of patients were diagnosed with significant MS. Therefore, prospective studies utilizing flow dependent measures of MS severity following MV repair and including a greater number of patients with iatrogenic MS are warranted to validate these results.

In conclusion, elevated mean and peak TMPGs obtained in the post-CPB period are practical and reliable indicators of significant MS immediately following MV repair. However, these values should not necessarily be considered pathognomonic for isolated perioperative MV dysfunction. Nonetheless, identifying an increased TMPG following MV repair should alert the intraoperative echocardiographer to consider acute MS especially in the presence of concurrent hemodynamic instability, and may allow the cardiac surgeon to consider a prompt revision prior to the development of significant postoperative morbidity and mortality. Further study is warranted to prospectively evaluate the impact of both intraoperative TEE Doppler derived gradient pressures and 3D TEE indices of MV area on perioperative surgical decision making in patients undergoing valve MV repair [47], [48].
